# Enhanced sensitivity, robust p21 activation, and sustained DNA repair responses to interstrand crosslinks in elephant cells compared to humans

**DOI:** 10.3389/fvets.2025.1570720

**Published:** 2025-07-10

**Authors:** Taisuke Kitano, Zida Zhu, Naoya Minami, Koichi Orino, Yasunaga Yoshikawa

**Affiliations:** Laboratory of Veterinary Biochemistry, School of Veterinary Medicine, Kitasato University, Aomori, Japan

**Keywords:** elephant, p21, FANCL, interstrand crosslink repair, comparative molecular biology, RAD51

## Abstract

Elephants exhibit remarkable resistance to cancer, and understanding these mechanisms has focused on their potential applications in cancer prevention and treatment in humans. A genome-wide comparative analysis identified that the accelerated regions in elephants are enriched in Fanconi anemia (FA) complementation group L (FANCL), a ubiquitin E3 ligase that mediates the monoubiquitylation of FANCD2 as an essential step in the FA pathway. The FA pathway plays a crucial role in DNA interstrand crosslink (ICL) repair, contributing substantially to genome stability and cancer resistance. In this study, we investigated the differences in ICL repair via the FA pathway, including the function of FANCL, as well as the DNA damage response to ICLs between elephants and humans. We found that elephant fibroblasts exhibited higher sensitivity to ICL-inducing treatments, such as mitomycin C and trimethylpsoralen plus UVA (PUVA), than human fibroblasts, while showing comparable or reduced sensitivity to other DNA-damaging agents, such as doxorubicin and bleomycin. Functional analyses revealed that elephant and human FANCL performed similarly in mediating FANCD2 monoubiquitylation and cell viability following mitomycin C treatment. Interestingly, elephant fibroblasts exhibited a more potent and prolonged activation of p21 and sustained DNA repair responses, such as FANCD2 monoubiquitylation and increased RAD51expression, following ICL-induced treatments. Moreover, elephant fibroblasts showed significantly greater RAD51 foci formation than human fibroblasts after PUVA treatment, even under comparable levels of DNA damage. These findings suggest that elephants efficiently repair ICLs in growth-arrested cells likely through robust p21 activation. This study provides new insights into the cancer resistance mechanisms of elephants and offers novel approaches for cancer prevention and therapy.

## 1 Introduction

Elephants are known for their remarkable resistance to cancer. Despite their large size and long lifespan, their cancer incidence and mortality rates are estimated to be < 5%, which is significantly lower than the >20% observed in humans (1, 2). The mechanism of cancer resistance in elephants has been the focus of much research because of its potential application in cancer prevention and treatment in humans (3). As a key mechanism, the elephant genome encodes 20 copies of the tumor suppressor gene *TP53*, which enhances the apoptotic response following DNA damage (1, 4). Additionally, p21 (Waf1/Cip1), a primary downstream target of p53, is strongly upregulated in elephant cells following DNA damage (1). Moreover, elephants possess additional leukemia inhibitory factor (LIF) genes such as *LIF6*, which are upregulated by p53 and further promote apoptosis in response to DNA damage (5). Thus, elephants have evolved mechanisms to resist cancer by activating systems that efficiently eliminate aberrant cells. Interestingly, a genome-wide comparative analysis of accelerated regions revealed that these regions in elephants were uniquely enriched near DNA damage response genes, and the top hotspot was observed in Fanconi anemia (FA) complementation group L (FANCL), a master regulator of the FA DNA repair pathway (6).

The FA pathway plays a crucial role in the repair of DNA interstrand crosslinks (ICLs), which are highly cytotoxic DNA lesions that interfere with critical cellular processes such as DNA replication and transcription (7). Importantly, ICLs can naturally arise within the body due to exposure to endogenous aldehydes generated during normal cellular metabolism as well as from exogenous aldehydes and environmental factors (8, 9). In the FA pathway, at least 22 FA proteins sequentially contribute to the repair of ICLs through distinct steps, including the removal or unhooking of ICLs (which generates DNA double-strand breaks) and subsequent repair via homologous recombination (10). In these steps, FANCL, which functions as a ubiquitin E3 ligase in the core complex with other FA proteins, mediates the monoubiquitylation of FANCD2 and FANCI, which constitutes an essential step in the FA pathway (11). Notably, cancer predisposition is one of the primary symptoms in patients with FA caused by homozygous germline mutations in the FA genes, and somatic mutations and copy number variations in these genes are frequently observed in various human cancers (12, 13). These findings highlight that the FA pathway plays a substantial role in maintaining genomic stability and is crucial for cancer resistance.

Based on these findings, we hypothesized that the characteristics of FANCL, along with the FA pathway and DNA damage responses to ICLs, contribute to cancer resistance mechanisms in elephants. However, limited research has focused on the FA pathway and DNA repair mechanisms in elephants. In this study, we aimed to investigate the differences in ICL repair via the FA pathway, including the function of FANCL, as well as the DNA damage responses to ICLs between elephants and humans as a step toward understanding their unique cancer resistance mechanisms.

## 2 Materials and methods

### 2.1 Chemicals and treatments

Mitomycin C (MMC; product no. 20898-21) was purchased from NACALAI TESQUE (Kyoto, Japan). Trimethylpsoralen (product no. T6137) was purchased from Merck (Darmstadt, Germany). Doxorubicin (DOX; product no. 15007) was purchased from Cayman Chemical (Ann Arbor, MI, USA). Bleomycin (product no. HY-17565) was purchased from MedChemExpress (Monmouth Junction, NJ, USA). All drugs were diluted in culture medium and applied to the cells. Psoralens plus UVA (PUVA) treatment was conducted by pre-incubation with trimethylpsoralen for 4 h followed by UVA (0.1 J/cm^2^, delivered over ~10 s) exposure. For Western blotting and immunostaining, the antibodies against FANCL (Product No. sc-137076), and p21 (product no. sc-271610) were purchased from Santa Cruz Biotechnology (Dallas, TX, USA). Antibodies against β-actin (product no. PM053), α-tubulin (product no. M175-3), and lamin B1 (product no. PM064) were purchased from MEDICAL and BIOLOGICAL LABORATORIES (Tokyo, Japan). Antibody against FANCD2 (product no. EPR2302) was purchased from Abcam (Cambridge, UK). Antibody against RAD51 (product no. 70-012) was purchased from BioAcademia (Osaka, Japan). Antibody against FLAG-tag (DYKDDDDK) (product no. KO602-L) was purchased from TransGenic (Tokyo, Japan). Antibody against phospho-histone H2A.X (Ser139) (γH2AX; product no. 05-636) was purchased from Merck.

### 2.2 Cell cultures and generation of cell lines

Fibroblasts isolated from the ear skin of African elephants (LACF-NaNaII) were provided by the RIKEN Cell Bank (Ibaraki, Japan) through the National BioResource Project of MEXT. Human oral mucosal fibroblasts (hOMF100) were purchased from the Cell Research Corporation (Singapore). Both cells were cultured in α-minimum essential medium (NACALAI TESQUE) supplemented with 10% fetal bovine serum (FBS). Human HeLa cells were purchased from the RIKEN Cell Bank and cultured in Dulbecco's modified Eagle's medium (FUJIFILM Wako Pure Chemical, Osaka, Japan) supplemented with 10% FBS. All cells were maintained at 37°C in a humidified incubator with 5% CO_2_.

The FANCL-knockout (KO) HeLa cells were generated by CRISPR-Cas9 using the gRNA sequence 5′-CGGTCGAAAACCGTGTATGA-3′ in the pSpCas9(BB)-2A-Puro (PX459) V2.0 vector (Addgene plasmid no. 190542). Exogenous expression of human or elephant FANCL in LACF-NaNaII and HeLa cells was achieved using retroviral transduction and magnetic sorting. Retroviruses were obtained from Phoenix A cells transfected with a pOZ-N vector carrying human or elephant FANCL. All plasmid DNA transfections were performed using FuGENE HD (Promega, Madison, WI, USA) according to the manufacturer's instructions.

### 2.3 Cell viability and proliferation assay

Cells were seeded into 96-well plates at 1,000 cells/well and incubated for 24 h. For cell viability assay, cells were treated with drugs for 96 h and then incubated with 10 μL of Cell Counting Kit-8 solution (DOJINDO, Kumamoto, Japan) for 3 h. Absorbance was measured at 450 nm using a microplate reader, and cell viability was calculated as the ratio of absorbance to non-treated cells. For the cell proliferation assay, at each time points of 24, 48, 96, and 120 h after seeding, the cells were incubated with Cell Counting Kit-8 solution for 3 h, and the absorbance was measured in the same way. The cell proliferation rate was calculated as the ratio of the absorbance of the cells at 24 h after seeding.

### 2.4 Western blotting

Cells were lysed in an equal volume of Benzonase buffer (20 mM Tris-HCl, pH 8.0; 2 mM MgCl_2_; 1% Triton X-100; 10% glycerol; and >12.5 U/ml Benzonase) at 4°C for 10 min and then treated with the same volume of 2% SDS to extract the total proteins. The lysates were mixed with 4 × LDS loading buffer (Thermo Fisher Scientific, Waltham, MA, USA) and 10 × reducing agent (Thermo Fisher Scientific). The samples were separated by 4–15% SDS-PAGE and transferred onto nitrocellulose membranes (Cytiva, Tokyo, Japan). The membranes were blocked with 5% skimmed milk and then incubated overnight at 4°C with the primary antibodies. Next, the membranes were incubated with horseradish peroxidase-conjugated secondary anti-mouse or anti-rabbit antibodies (MEDICAL & BIOLOGICAL LABORATORIES) for 1 h at room temperature. Immunoreactive bands were visualized using Immunostar Zeta or Immunostar LD (for higher-sensitivity detection) (FUJIFILM Wako Pure Chemical) and detected using a C-DiGit Blot Scanner (LI-COR, Lincoln, NE, USA). Band intensities were measured using the Image Studio Software (LI-COR).

### 2.5 Immunostaining and image analysis

Cells were cultured on coverslips (Matsunami Glass, Osaka, Japan) and treated with PUVA (0.1 μg/mL TMP). At 0, 3, 6, 12, 24, and 48 h after treatment, cells were fixed with 4% paraformaldehyde for 5 min at room temperature. Following permeabilization with phosphate-buffered saline (PBS) containing 1% Triton X-100 for 10 min, cells were incubated with PBS containing 5% bovine serum albumin (BSA) at room temperature for 1 h. Subsequently, cells were incubated overnight at 4°C with anti-γH2AX and anti-RAD51 antibodies diluted in PBS with 5% BSA. After washing, cells were stained for 1 h at room temperature with Alexa Fluor 488-conjugated goat anti-mouse IgG and Alexa Fluor 568-conjugated goat anti-rabbit IgG (Thermo Fisher Scientific), along with 4′,6-diamidino-2-phenylindole (DAPI; Thermo Fisher Scientific), all diluted in PBS containing 5% BSA. Coverslips were mounted using VECTASHIELD^®^ Antifade Mounting Medium (Vector Laboratories, Newark, CA, USA). Cells were imaged using a confocal laser scanning microscope (Carl Zeiss AG, Oberkochen, Germany) equipped with ZEN software (Carl Zeiss AG). Nuclear γH2AX and RAD51 foci were analyzed in >70 cells per group using Cellpose3 (14) and ImageJ software (National Institutes of Health, Bethesda, MD, USA). Briefly, individual nuclei were segmented based on DAPI staining using Cellpose3, and the resulting outlines were used as nuclear regions of interest (ROIs) for subsequent analyses. Foci in the γH2AX and RAD51 channels were identified by subtracting a Gaussian-blurred background image, followed by local auto-thresholding using the “Bernsen” method and conversion to binary masks. The area fraction (%) of γH2AX and RAD51 foci was quantified within each nuclear ROI. Additionally, the percentage of RAD51-positive area within γH2AX foci was calculated for nuclei with a γH2AX area fraction >1.5%.

### 2.6 Data analysis

Data are expressed as mean ± standard error of the mean (SEM) (*n* = number of independent experiments). Two-group comparisons were performed using Welch's *t*-tests or the exact Wilcoxon rank-sum test (also known as the Mann-Whitney U test). Multiple comparisons were performed using one-way ANOVAs followed by Tukey's HSD test, Dunnett's test, or Steel's test (with control group), as appropriate. Statistical significance was set at *p* < 0.05. All statistical analyses were performed using the JMP Pro software (SAS, Cary, NC, USA). Comparisons between the control and treatment, as well as between elephant and human cells, were analyzed based on independent hypotheses; therefore, no correction was applied. All graphs were constructed using Excel (Microsoft, Redmond, WA, USA).

## 3 Results

### 3.1 Elephant fibroblasts exhibited higher sensitivity to ICL-inducing treatments compared to human fibroblasts

**Figure 1:**
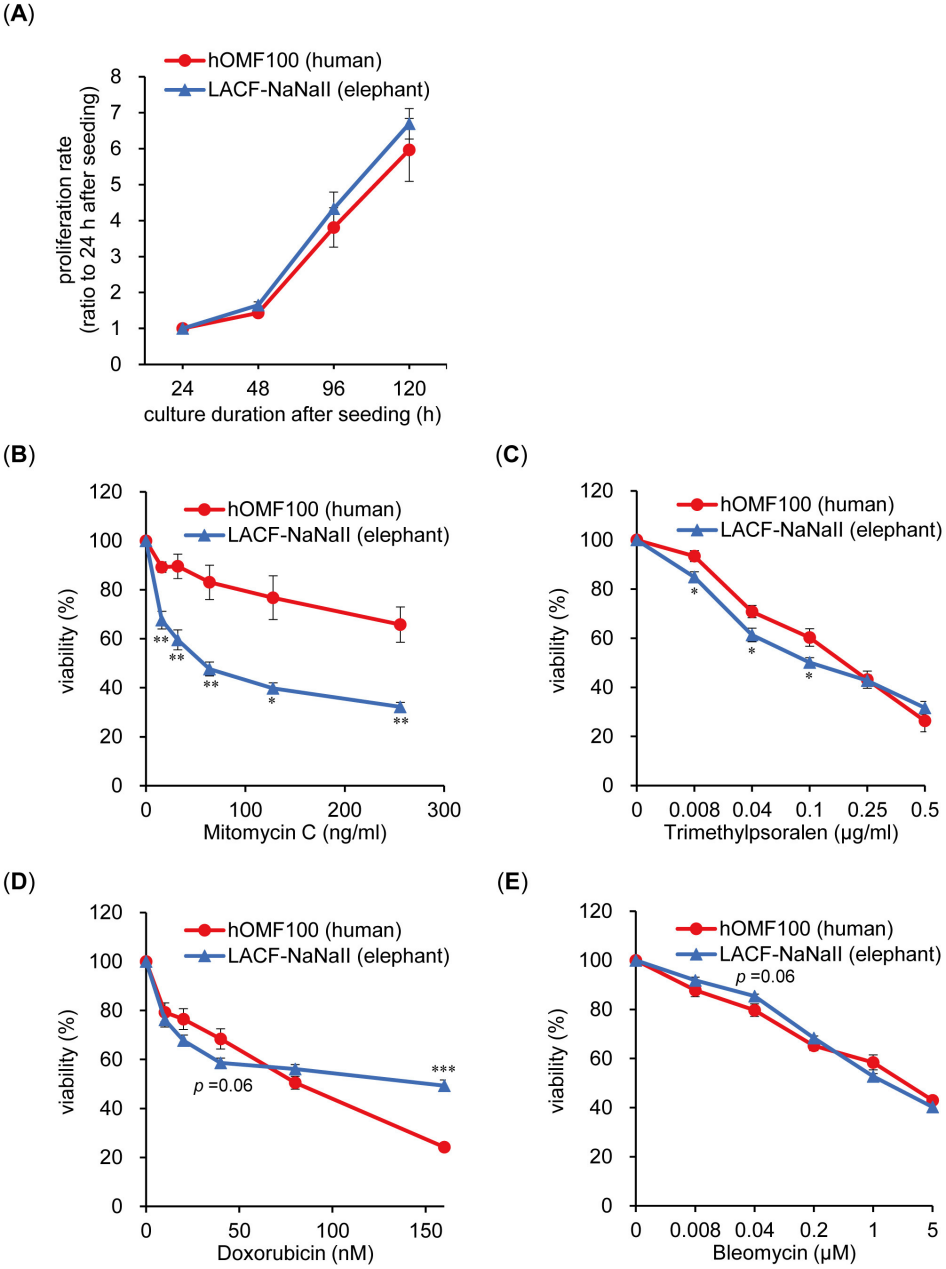
Elephant fibroblasts exhibit higher sensitivity to DNA ICL treatments compared to human fibroblasts. **(A)** Proliferation rate of hOMF100 and LACF-NaNaII cells at 24–120 h after seeding (*n* = 6–9). **(B**–**E)** Viability of hOMF100 and LACF-NaNaII cells at different concentrations of mitomycin C (*n* = 5) **(B)**, trimethylpsoralen (plus UVA [0.1 J/cm^2^]: PUVA) (*n* = 6) **(C)**, doxorubicin (*n* = 7) **(D)** or bleomycin (*n* = 9) **(E)**. *p < 0.05, **p < 0.01 vs. hOMF100 cells (Welch's *t*-tests). All data are presented as mean ± SEM.

To compare the properties in the viability to ICLs between human and elephant cells, we examined the effects of ICL-induced treatment on the viability of human and elephant cells. Human (hOMF100) and African elephant (LACF-NaNaII) fibroblasts were used because these cells exhibit comparable proliferative capacities under identical culture conditions (Figure 1A). MMC, a major DNA crosslinking agent, reduced the viability of LACF-NaNaII cells in a dose-dependent manner, with a significantly greater effect than on hOMF100 cells (Figure 1B). A similar differential sensitivity to MMC was also observed in the clonogenic assay (Supplementary Figure S1). Moreover, LACF-NaNaII cells exhibited a higher sensitivity to trimethylpsoralen plus UVA (PUVA) treatment, which induces ICLs more effectively and specifically (15) (Figure 1C). Consistent with these findings, cell cycle analysis revealed a pronounced G2/M arrest in LACF-NaNaII cells following MMC treatment (Supplementary Figure S2). In contrast, this increased sensitivity in LACF-NaNaII cells was not observed with other DNA-damaging agents such as DOX, which intercalates into the DNA and inhibits topoisomerase II, or bleomycin, which induces DNA double-strand breaks (Figures 1D, E). In contrast, LACF-NaNaII cells exhibited lower sensitivity to higher DOX concentrations (160 nM). These results suggest that elephant cells are more sensitive to ICLs than are human cells.

### 3.2 Comparable function of elephant and human FANCL in ICL repair

*FANCL* locus has been identified as a genomic hotspot of elephant-accelerated regions (6). Elephant genomic DNA information has been reported, but the sequence of *FANCL* mRNA has not been confirmed. Therefore, we first determined the full-length elephant FNACL cDNA (Accession No. LC858708). The open reading frame consisted of 1,119 base pairs, and the predicted protein length was 372 amino acids, which was three amino acids shorter than that of human FANCL. Moreover, amino acid sequence alignment between elephant and human FANCL revealed three substitutions in elephant FANCL at functionally significant positions: I136F, F252R, and I265L (Supplementary Figure S3). These residues are critical for FANCL folding or the monoubiquitylation of FANCD2, as reported in studies on human FANCL mutations (16). Thus, we investigated the functional differences between elephant and human FANCL and their potential relationship with the enhanced sensitivity of elephant cells to ICLs.

**Figure 2:**
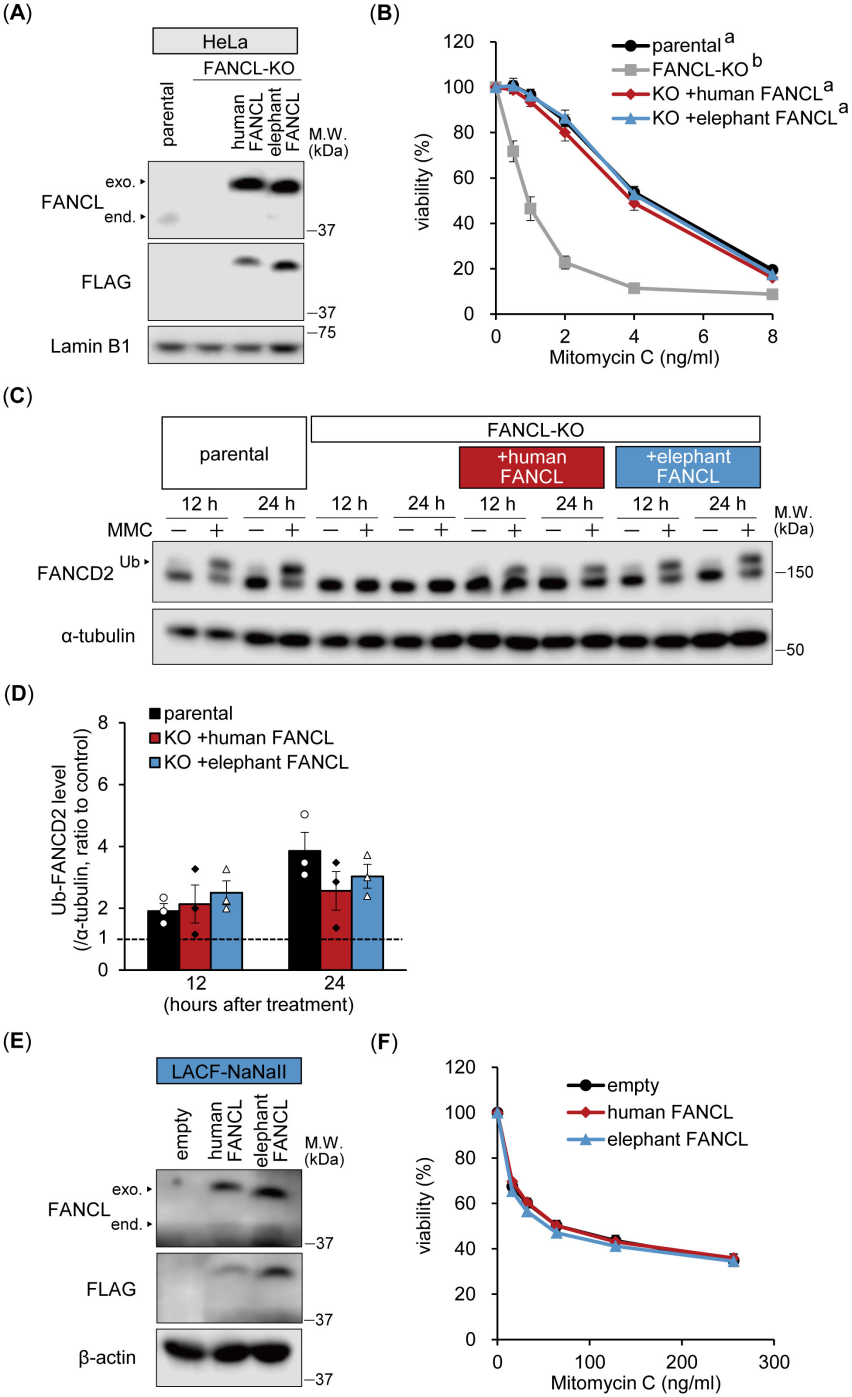
Functional comparison between human and elephant FANCL. **(A, B)** Expression levels of endogenous (end.) FANCL and exogenous (exo.) FLAG-HA-fused human or elephant FANCL in four types of HeLa cells: parental, FANCL knockout (KO), and FANCL-KO cells expressing exogenous human or elephant FANCL, were analyzed via Western blotting using anti-FANCL and FLAG antibodies **(A)**. Viability of these cells at different concentrations of mitomycin C (MMC) was examined (*n* = 5) **(B)**. Different letters indicate statistically significant differences (*p* < 0.01, Tukey's HSD test) at all concentrations. **(C**, **D**) Monoubiquitylation (Ub) of FANCD2 at 12 or 24 h after treatment with MMC (160 ng/mL) in parental, FANCL-KO HeLa cells, and FANCL-KO cells expressing exogenous human or elephant FANCL, were evaluated via Western blotting using anti-FANCD2 antibody **(C)**. Change ratio of Ub-FANCD2 levels with MMC treatment were quantified (*n* = 3) **(D). (E, F)** Expression levels of endogenous FANCL and exogenous FLAG-HA-fused human or elephant FANCL in LACF-NaNaII cells, transduced with empty vector or vector carrying human or elephant FANCL, were observed via Western blotting using anti-FANCL and FLAG antibodies **(E)**. Viability of these cells at different concentrations of MMC was examined (*n* = 6) **(F)**. All data are presented as mean ± SEM with or without individual data points.

To compare FANCL functions, we generated FANCL-KO HeLa cells and reconstituted them with either human or elephant FANCL (Figure 2A). FANCL-KO cells exhibited significantly increased sensitivity to MMC, which was restored to a similar extent by exogenous expression of both human and elephant FANCL (Figure 2B). Furthermore, we examined the functional differences between human and elephant FANCL in the monoubiquitylation of FANCD2 after treatment with MMC (160 ng/mL) for 12 or 24 h (Figure 2C). The change in the ratio of monoubiquitylated FANCD2 levels was not significantly different between parental cells and cells expressing human or elephant FANCL (Figure 2D). Nuclear FANCD2 foci formation after MMC treatment was also restored in these cells (Supplementary Figure S4). These results indicate that the function of elephant FANCL in ICL repair is comparable to that of human FANCL when expressed in human cells. To investigate whether higher expression levels of FANCL rescued the enhanced sensitivity of elephant cells to ICLs, we generated LACF-NaNaII cells expressing exogenous human or elephant FANCL (Figure 2E). MMC sensitivity was not influenced by exogenous expression of either human or elephant FANCL (Figure 2F), suggesting that the heightened sensitivity of elephant cells to ICLs is not attributable to differences in FANCL function or expression levels.

### 3.3 Elephant fibroblasts exhibit a potent p21 response to ICL-inducing agents compared to human fibroblasts

**Figure 3:**
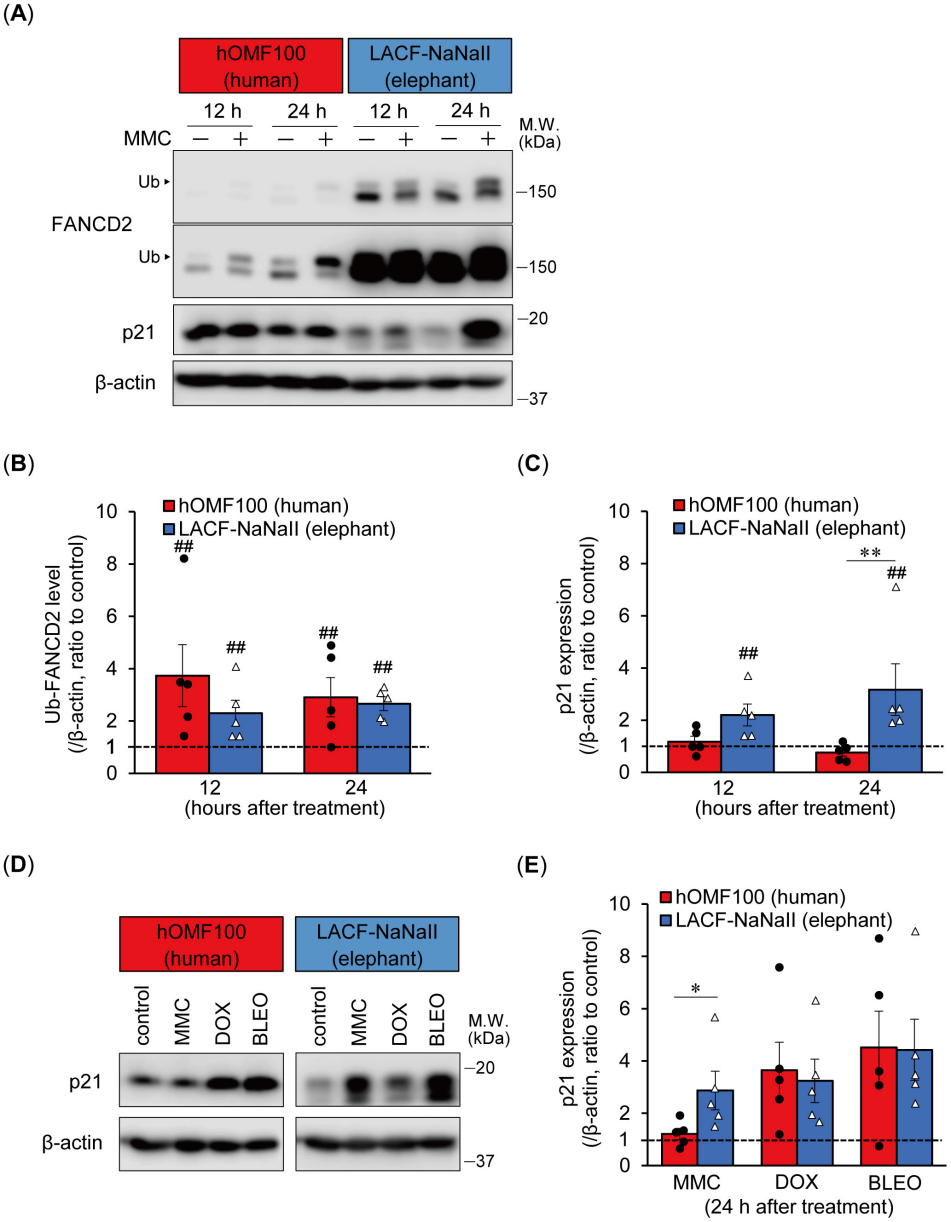
Elephant fibroblasts exhibit potent p21 response to DNA ICL agent. **(A**–**C)** Monoubiquitylation (Ub) of FANCD2 and p21 expression levels at 12 or 24 h after treatment with mitomycin C (MMC; 256 ng/mL) in hOMF100 and LACF-NaNaII cells were observed via Western blotting using anti-FANCD2 and p21 antibodies **(A)**. Change ratio of Ub-FANCD2 levels **(B)** and p21 expression levels **(C)** with MMC treatment were quantified (*n* = 5). Two images of same membrane with different contrast settings for FANCD2 are shown. ^##^*p* < 0.01, control vs. MMC treatment (exact Wilcoxon rank-sum test). **p < 0.01, hOMF100 vs. LACF-NaNaII cells (exact Wilcoxon rank-sum test). **(D, E)** p21 expression levels at 24 h after treatment of MMC (256 ng/mL), doxorubicin (DOX; 80 nM), or bleomycin (BLEO; 5 μM) in hOMF100 and LACF-NaNaII cells were analyzed via Western blotting using anti-p21 antibody **(D)**. Change ratio of p21 expression with drug treatments were quantified (*n* = 5) **(E)**. *p < 0.05, hOMF100 vs. LACF-NaNaII cells (exact Wilcoxon rank-sum test). All data are presented as mean ± SEM with individual data points.

Elephant fibroblasts exhibit increased p21 protein expression and reduced viability following ionizing radiation exposure compared to human fibroblasts (1). To investigate the mechanism underlying the higher sensitivity of elephant cells to ICL treatment, we compared the ICL damage response following MMC treatment of hOMF100 and LACF-NaNaII cells (Figure 3A). Treatment with MMC (256 ng/mL) for 12 and 24 h enhanced the monoubiquitylation of FANCD2 in both cell lines. Higher levels of FANCD2 were detected in LACF-NaNaII cells than in hOMF100 cells. Additionally, even after MMC treatment, the proportion of non-ubiquitylated FANCD2 relative to ubiquitylated FANCD2 was higher in LACF-NaNaII cells than in hOMF100 cells. The change in the ratio of monoubiquitylated FANCD2 following MMC treatment was not significantly different between these cells (Figure 3B), suggesting a comparable ICL repair response between elephant and human cells. Interestingly, treatment with MMC for 24 h increased p21 expression in LACF-NaNaII cells but not in hOMF100 cells (Figure 3C). In contrast, DOX (80 nM) and bleomycin (5 μM) increased p21 expression to the same level in both cell lines (Figures 3D, E), consistent with their comparable effects on cell viability, as shown in Figure 1. These results suggest that ICLs induce a more potent p21 response in elephant cells than in human cells.

### 3.4 Prolonged and robust p21 activation and sustained DNA repair responses following ICL-inducing treatment in elephant fibroblasts

**Figure 4:**
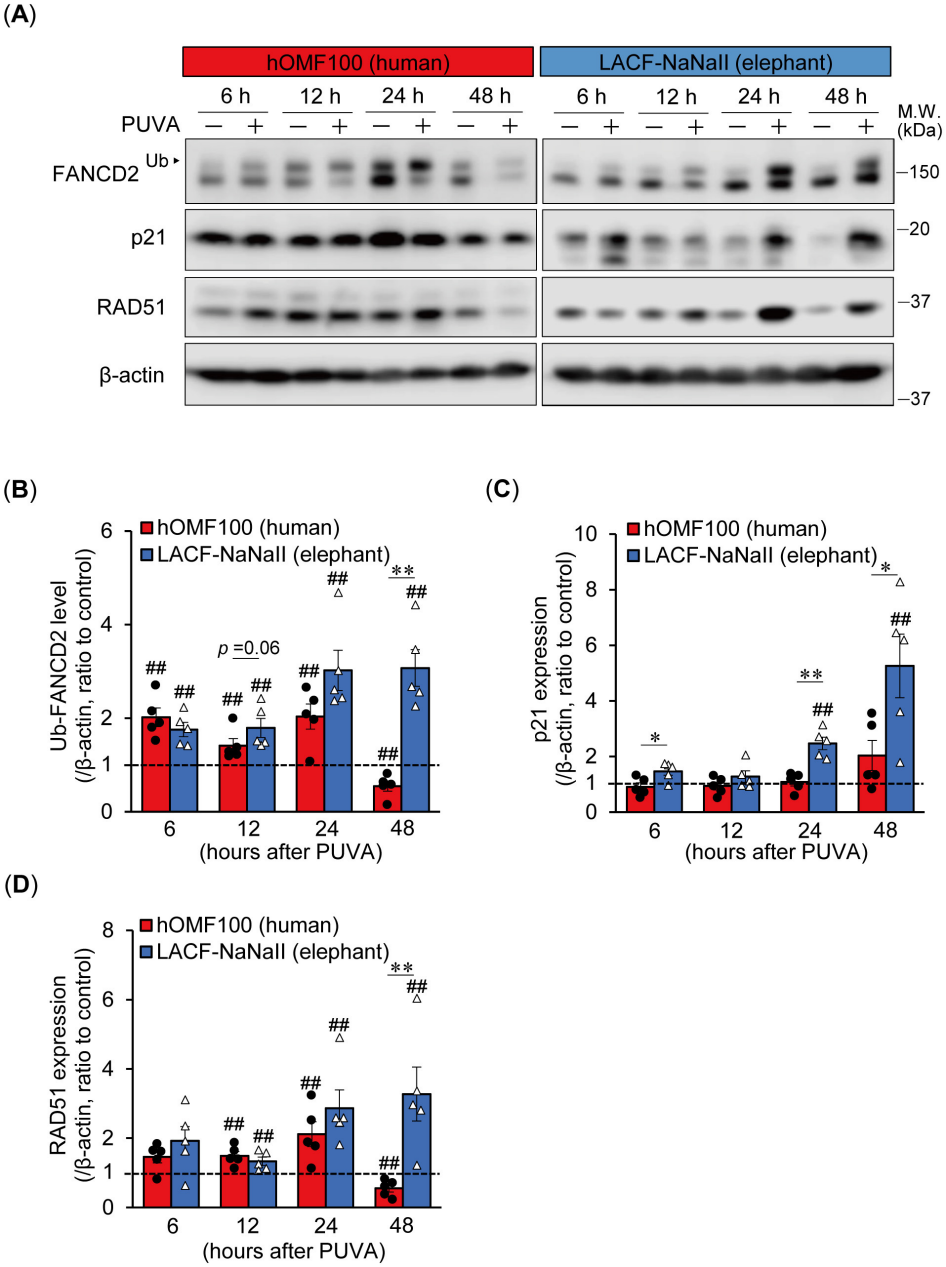
Elephant fibroblasts exhibit prolonged and robust p21 activation along with sustained DNA repair responses following ICL treatment compared to human fibroblasts. **(A–D)** Monoubiquitylation (Ub) of FANCD2 and expression levels of p21 and RAD51 at 6, 12, 24, or 48 h after treatment with trimethylpsoralen (0.1 μg/mL) plus UVA (0.1 J/cm^2^) (PUVA) in hOMF100 and LACF-NaNaII cells were analyzed via Western blotting using anti-FANCD2, p21, and RAD51 antibodies **(A)**. Change ratio of Ub-FANCD2 levels **(B)** and expression levels of p21 **(C)** and RAD51 **(D)** with PUVA treatment were quantified (*n* = 5). ^##^*p* < 0.01, control vs. PUVA treatment (exact Wilcoxon rank-sum test). *p < 0.05, **p < 0.01, hOMF100 vs. LACF-NaNaII cells (exact Wilcoxon rank-sum test). All data are presented as mean ± SEM with individual data points.

**Figure 5:**
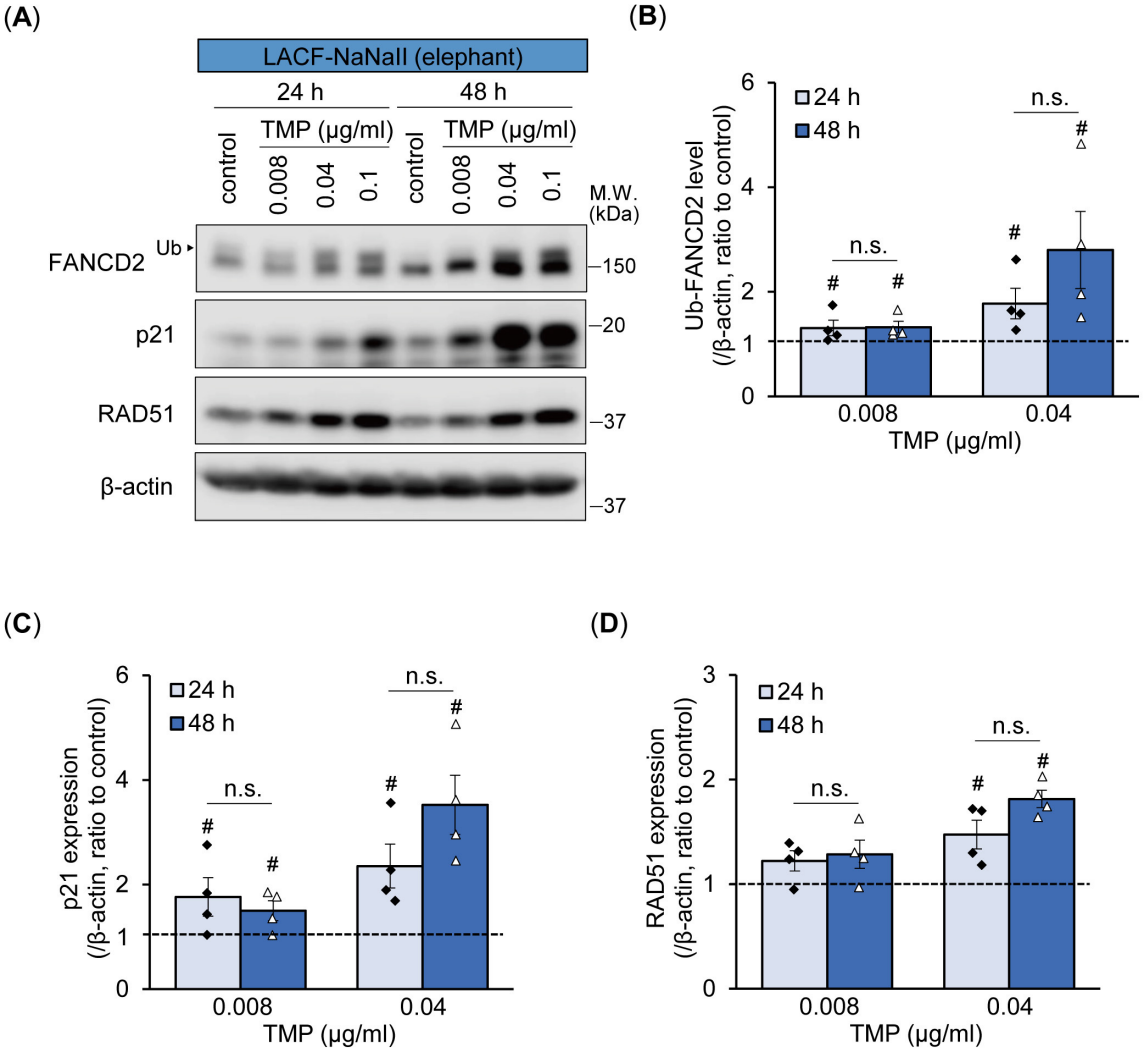
Prolonged p21 and sustained DNA repair responses in elephant fibroblasts appear to be independent of ICL levels. **(A–D)** Monoubiquitylation (Ub) of FANCD2 and expression levels of p21 and RAD51 at 24 or 48 h after treatment with different concentrations of trimethylpsoralen (TMP) plus UVA (0.1 J/cm^2^) (PUVA) in LACF-NaNaII cells were evaluated via Western blotting using anti-FANCD2, p21, and RAD51 antibodies **(A)**. Change ratio of Ub-FANCD2 levels **(B)** and expression levels of p21 **(C)** and RAD51 **(D)** with PUVA treatment were quantified (n = 4). ^#^*p* < 0.05 vs. control (Steel's test). Not significant (n.s.), 24 h vs. 48 h after PUVA treatment (exact Wilcoxon rank-sum test). All data are presented as mean ± SEM with individual data points.

We further investigated time-dependent differences in the monoubiquitylation of FANCD2 and p21 expression between human and elephant cells following ICL induction using PUVA treatment, which induces ICLs more specifically at the precise time of UVA exposure (Figure 4A). In addition to FANCD2 and p21, we also examined the expression of RAD51 recombinase (also known as FANCR), which is essential for homologous recombination following the removal of ICLs during ICL repair (17). PUVA treatment (trimethylpsoralen [0.1 μg/mL] plus UVA [0.1 J/cm^2^]) enhanced the monoubiquitylation of FANCD2 in both cells starting 6 h after treatment. In LACF-NaNaII cells, even after PUVA treatment, the proportion of non-ubiquitylated FANCD2 relative to ubiquitylated FANCD2 was higher than that in hOMF100 cells. Interestingly, increased levels of monoubiquitylated FANCD2 following PUVA treatment were sustained for up to 48 h in LACF-NaNaII cells; however, they were not sustained and instead decreased in hOMF100 cells (Figure 4B). Consistent with the results of MMC treatment, an increase in p21 expression was observed in LACF-NaNaII cells following PUVA treatment, starting at 24 h post-treatment and persisting for up to 48 h, whereas no such increase was observed in hOMF100 cells (Figure 4C). Moreover, PUVA treatment increased RAD51 expression in both cell lines starting at 12 h after treatment. Similar to monoubiquitylated FANCD2, this increase was sustained for up to 48 h post-treatment in LACF-NaNaII cells, and decreased by 48 h in hOMF100 cells (Figure 4D). These results indicate that ICLs induce a more prolonged and robust activation of p21, along with sustained DNA repair responses, in elephant cells than in human cells. To investigate whether these long-lasting responses to ICLs in elephant cells are dependent on the level of ICLs, specifically whether the prolonged responses result from higher levels of ICLs, we performed PUVA treatment using lower concentrations (0.04 and 0.008 μg/ml) of trimethylpsoralen and evaluated the responses in LACF-NaNaII cells (Figure 5A). These concentrations exerted similar or less effects on the viability of LACF-NaNaII cells compared to the effect of 0.1 μg/ml trimethylpsoralen on hOMF100 cells. PUVA treatment increased the levels of monoubiquitylated FANCD2 (Figure 5B), p21 (Figure 5C), and RAD51 (Figure 5D) in a dose-dependent manner 24 h post-treatment. These responses were sustained by 48 h. Together, these results suggest that prolonged p21 activation and sustained DNA repair responses in elephant cells are independent of ICL levels.

### 3.5 Enhanced DNA repair responses under comparable levels of DNA damage after ICL-inducing treatment in elephant fibroblasts

**Figure 6:**
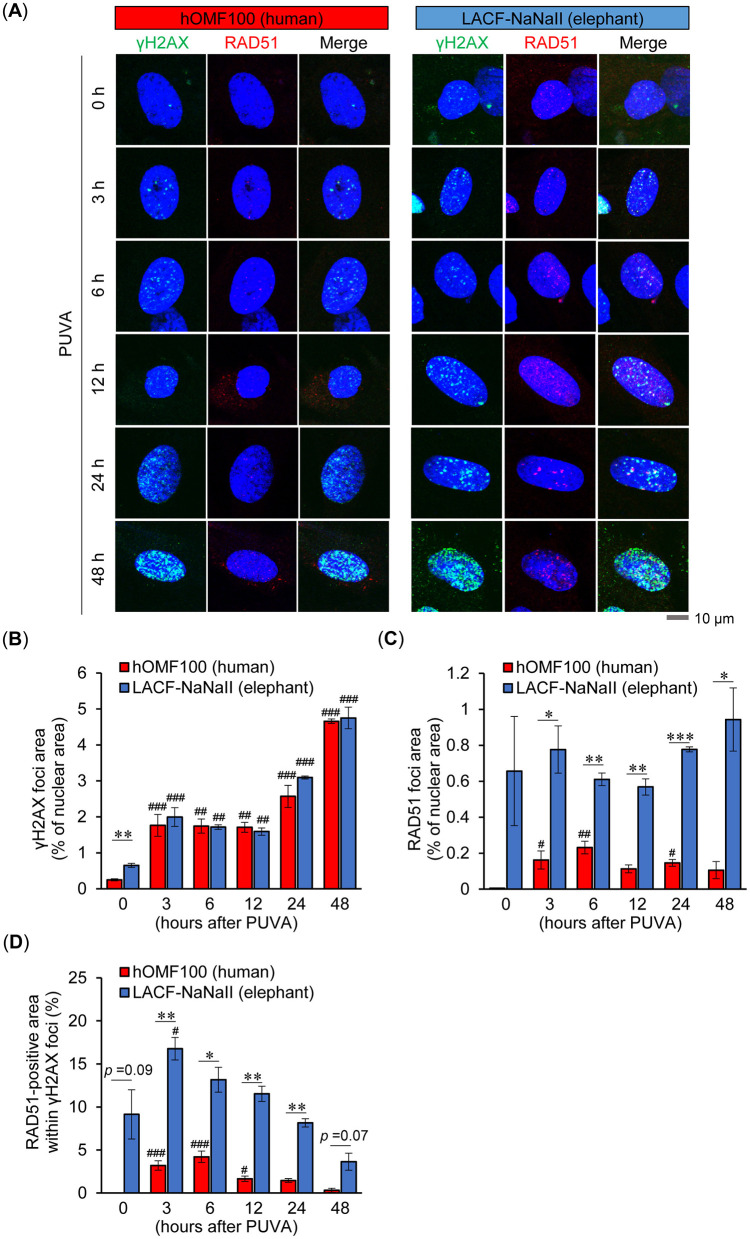
Elephant fibroblasts exhibit higher RAD51 foci formation than human fibroblasts under comparable levels of DNA damage. **(A)** Formation of γH2AX (green) and RAD51 (red) foci in DAPI-stained nuclei (blue) at 0, 3, 6, 12, 24 and 48 h after treatment with trimethylpsoralen (0.1 μg/mL) plus UVA (0.1 J/cm^2^) (PUVA) in hOMF100 and LACF-NaNaII cells were evaluated via immunostaining. Representative images of a single cell at each timepoint are shown. Scale bar = 10 μm. **(B–D)** Percentage of γH2AX **(B)** and RAD51 **(C)** foci area in nuclei, and RAD51-positive area within γH2AX foci **(D)** were quantified (*n* = 3). ^#^*p* < 0.05, ^##^*p* < 0.01, ^###^*p* < 0.001 vs. 0 h (Dunnett's test). *p < 0.05, **p < 0.01, ***p < 0.001, hOMF100 vs. LACF-NaNaII cells (Welch's *t*-tests). All data are presented as mean ± SEM.

To elucidate the differences in DNA damage and repair responses—and their relationship—following ICL induction between human and elephant cells, we further examined the time-dependent formation of nuclear γH2AX foci, a highly sensitive marker of ICL-associated DNA damage (18, 19), as well as RAD51 foci following PUVA treatment (Figure 6A). Although the basal level of γH2AX foci was higher in LACF-NaNaII cells than in hOMF100 cells, PUVA treatment induced a continuous increase in γH2AX foci formation up to 48 h post-treatment to a similar extent in both cells (Figure 6B). These results suggest that PUVA treatment causes comparable levels of DNA damage in both human and elephant fibroblasts. RAD51 foci also tended to be higher at the basal level in LACF-NaNaII cells (Figure 6C). Although PUVA treatment induced RAD51 foci formation in hOMF100 cells, the levels at all time points remained significantly lower than those in LACF-NaNaII cells. Furthermore, the RAD51-positive area within γH2AX foci was (tended to be) higher in LACF-NaNaII cells compared to hOMF100 cells across all time points (Figure 6D). These findings suggest that elephant cells may exhibit a more robust RAD51-mediated DNA repair response under comparable levels of DNA damage.

## 4 Discussion

In this study, we observed that elephant cells exhibited enhanced sensitivity, prolonged and robust p21 activation, and sustained DNA repair responses (monoubiquitylation of FANCD2, increased expression of RAD51 and prominent formation of nuclear RAD51 foci), specifically following ICL-inducing treatment, compared to human cells. The comparable induction of nuclear γH2AX foci in both species following ICL-inducing treatment suggests that the differences in cellular responses are not due to differences in the extent of DNA damage. Furthermore, the similar functions of elephant and human FANCL suggest that their function is not likely to contribute to these differences.

Our results are consistent with those of a previous report that showed greater p21 upregulation following irradiation in elephant fibroblasts than in human fibroblasts (1). p21 is a critical mediator of cell-cycle arrest and is upregulated by p53 activation in response to DNA damage (20). Considering previous reports of more robust p53 signaling in elephant cells after DNA damage (1, 4), it is speculated that ICL-induced treatments strongly activate the p53-p21 pathway in elephant cells, leading to a more pronounced reduction in cell viability than in human cells. However, the specificity of the enhanced p21 response to ICLs in elephant cells may not be solely explained by the p53 mechanism, as enhanced p53 signaling in elephant fibroblasts has also been observed in response to other DNA-damaging treatments, such as DOX and UVC, in addition to MMC (although not directly compared with human fibroblasts) (4). Several studies have identified p53-independent mechanisms for the upregulation of p21, notably involving breast cancer susceptibility gene 1 (BRCA1, also known as FANCS), which functions as a component of the FA pathway (20, 21). In addition, increased p21 expression in FA pathway-deficient cells (with knockdown of FANCA or FANCD2) is partially mediated by microphthalmia-associated transcription factor and nucleophosmin 1 in a p53-independent manner (22). Unfortunately, in this study, we were unable to evaluate the expression of elephant p53 because it was not detected by western blotting using various commercially available anti-p53 antibodies (DO-1, DO-7, A-1, and C-11) (data not shown). The relationship between p53 signaling and enhanced p21expression in elephant cells in response to ICLs should be addressed in future studies.

In contrast to sustained FANCD2 monoubiquitylation and a sustained increase in RAD51 expression in elephant cells following ICL induction, these DNA repair responses disappeared 48 h after induction in human cells. Both FANCD2 and RAD51 are cleaved by caspase 3 and undergo proteolytic degradation during DNA damage-induced apoptosis (23–25). Additionally, p53 downregulates FA proteins, including FANCD2 and RAD51, by activating p21 (26). These mechanisms are likely to contribute to the reduction in the expression of monoubiquitylated FANCD2 and RAD51 following ICL induction in human cells. However, it seems unlikely that p53 signaling and the apoptotic response were strongly induced in human cells compared to elephant cells, given the lower sensitivity of human fibroblasts to PUVA in terms of viability, and previous reports indicate higher p53 signaling and caspase 3/7 activity in elephant cells in response to DNA damage (1, 4). Negative regulatory mechanisms affecting the expression of FANCD2 and RAD51 may be less active in elephant cells. Suzuki et al. have reported that p21 mediates the inactivation of caspase 3 (27, 28). Furthermore, p21 promotes MMC-induced FANCD2/I monoubiquitylation, likely through the transcriptional repression of the USP1, a deubiquitylating enzyme for FANCD2/I (29). Thus, the prolonged and robust activation of p21 following ICL induction may contribute to the sustained monoubiquitylation of FANCD2 and a sustained increase in RAD51 expression in elephant cells. Higher levels of FANCD2 were detected in elephant cells than in human cells. Additionally, even after ICL-inducing treatments, the proportion of non-ubiquitylated FANCD2 relative to ubiquitylated FANCD2 in elephant cells remained higher than that in human cells, despite a similar induction ratio of ubiquitylated FANCD2 in both cells. These observations suggest that elephant cells exhibit a higher overall expression of FANCD2, which may contribute to the sustained levels of monoubiquitylated FANCD2 following ICL induction. RAD51 plays a crucial role in homologous recombination and is essential for ICL repair (17). Monoubiquitylated FANCD2 promotes homologous recombination during ICL repair by interacting with CtIP, which is involved in DNA end resection (30–32). Moreover, regardless of monoubiquitylation, FANCD2 plays a crucial role in the stabilization of RAD51 in single-stranded DNA and promotes strand exchange activity, an important process in homologous recombination (33). Given this role, the higher expression of FANCD2 in elephant cells may partly account for the elevated formation of RAD51 foci. Taken together, sustained FANCD2 monoubiquitylation, along with the prolonged increase in RAD51 expression and nuclear foci formation in elephant cells, likely contribute to the error-free repair of ICLs and maintenance of genome stability. However, further investigations are needed to determine whether homologous recombination or other error-free repair pathways are indeed more active in elephant cells, and how these mechanisms influence cellular fate following ICL-induced DNA damage.

Our findings suggest that elephant cells may not only indiscriminately eliminate abnormal cells through apoptosis mediated by potent p53 signaling but also exhibit a remarkable ability to arrest cell proliferation via robust p21 signaling (likely regulated by p53), enabling efficient repair of DNA damage. Supporting this hypothesis, previous studies have demonstrated that p21 expression is positively associated with RAD51 expression and foci formation (34), and that p21 promotes error-free replication-coupled repair of DNA double-strand breaks (35). Although further research is necessary to confirm whether our findings are universally applicable to elephant cells, this strategy may explain the exceptional cancer resistance of elephants, allowing them to sustain numerous cells in their large bodies over a long lifespan. The modulation of p53 signaling by various mechanisms, depending on the type and extent of DNA damage, determines the cell fate, including apoptosis, cell cycle arrest, and DNA repair (36). Understanding how p53 signaling orchestrates these cell fates in elephants is particularly intriguing and may hold the key to uncovering the molecular basis of cancer resistance. This study provides new insights into the cancer resistance mechanisms of elephants, potentially offering novel approaches for cancer prevention and therapy in humans.

## Data Availability

The datasets presented in this study can be found in online repositories. The names of the repository/repositories and accession number(s) can be found in the article/Supplementary Material.
