# Supplementary material for: Enhanced sensitivity, robust p21 activation, and sustained DNA repair responses to interstrand crosslinks in elephant cells compared to humans
Source: Front Vet Sci. 2025 Jul 10;12:1570720. doi: 10.3389/fvets.2025.1570720 (PMC12287702; doi:10.3389/fvets.2025.1570720)
Supplement: Supplementary file 1 [file Data_Sheet_1.docx]

# Supplementary Materials and methods

## Clonogenic assay

Cells were seeded into 6-well plates at 200–800 cells/well and treated with 2–32 ng/mL mitomycin C on the following day. Two weeks after treatment, cell colonies were stained with 1% (w/v) crystal violet in methanol, then solubilized in 0.1% sodium citrate in 50% ehanol. Absorbance of the lysates was measured at 595 nm using a microplate reader, and cell viability was calculated as the ratio of absorbance to non-treated cells.

## Cell cycle analysis using flow cytometry

Cells treated with mitomycin C (25 and 50 ng/mL for 20 h) were harvested with cold PBS, fixed in 40% ethanol at 4°C, and stained with a propidium iodide (PI) solution (PBS containing 25 µg/mL PI [Thermo Fisher Scientific], 100 µg/mL RNase A, 0.1% Triton X-100, and 5% glycerol). The samples were analyzed using a CytoFLEX flow cytometer (Beckman Coulter Life Sciences, Indianapolis, IN, USA), and cell cycle distribution was assessed using FlowJo software (Becton, Dickinson and Company, Franklin Lakes, NJ, USA).


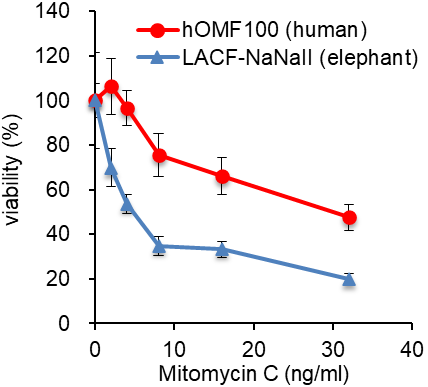


**

*

**

*

**

**Supplementary Figure S1.** Elephant fibroblasts exhibit higher sensitivity to DNA ICL treatments compared to human fibroblasts. Viability of hOMF100 and LACF-NaNaII cells was assessed by clonogenic assay after treatment with different concentrations of mitomycin C. **p* < 0.05, ***p* < 0.01 vs. hOMF100 cells (Welch's *t*-tests). All data are presented as mean ± standard deviation from three biological replicates (n = 3).


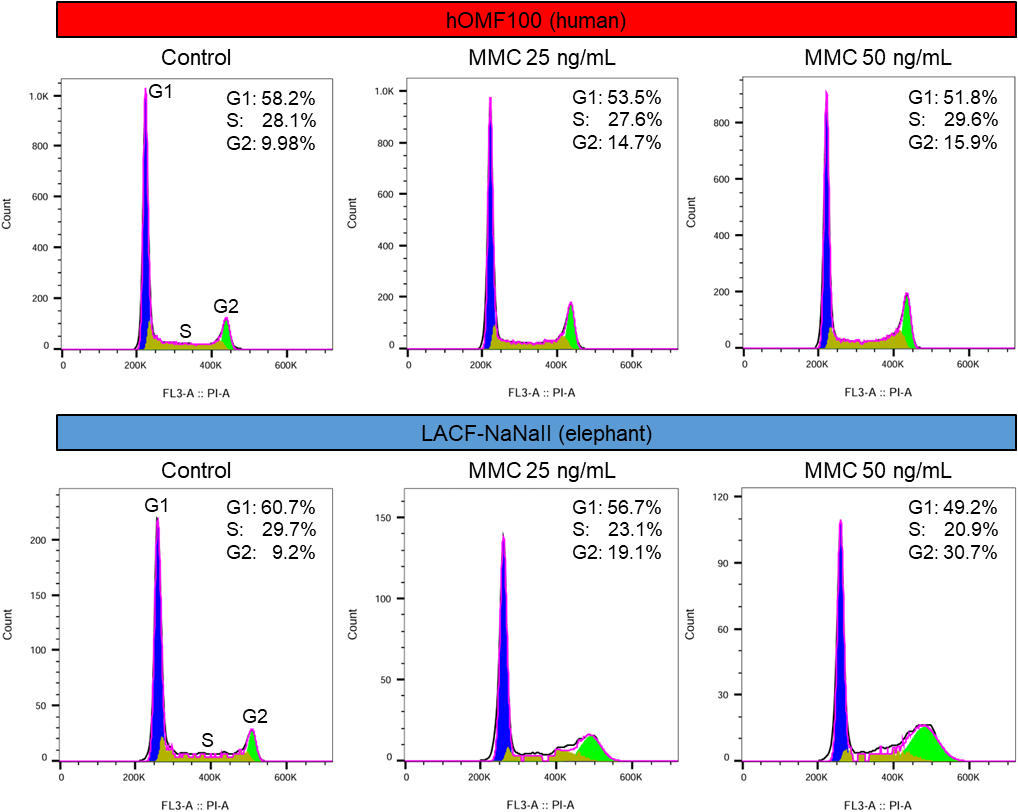


**Supplementary Figure S2.** Elephant fibroblasts exhibit more pronounced G2/M phase cell cycle arrest following DNA ICL treatments compared to human fibroblasts. Flow cytometry histograms show the distribution of hOMF100 and LACF-NaNaII cells treated with mitomycin C (MMC; 25 and 50 ng/mL for 20 h) across cell cycle phases. Percentages for each cell cycle phase are indicated in the upper right corner of each histogram. Similar histograms were observed in three independent biological replicates.

**Supplementary Figure S3.** The amino acid sequence alignment between human (*Homo sampiens*) and African elephant (*Loxodonta africana*) FANCL. The alignment was performed using BLAST (<https://blast.ncbi.nlm.nih.gov/Blast.cgi>, accessed on 4 December 2024). Asterisks indicate the difference between human and elephant, with important amino acids for the folding of FANCL or mono-ubiquitylation of FANCD2 highlighted by bold black boxes.


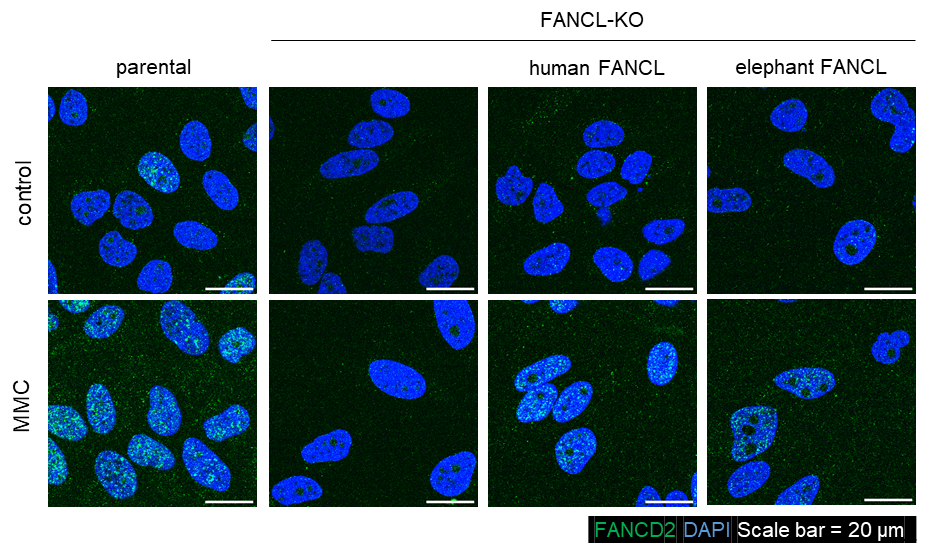


**Supplementary Figure S4.** Formation of nuclear FANCD2 foci in response to a DNA ICL agent. Nuclear FANCD2 foci were analyzed by immunostaining with an anti-FANCD2 antibody and DAPI staining after treatment with mitomycin C (MMC; 160 ng/mL for 16 h) in four types of HeLa cells: parental, FANCL knockout (KO), and FANCL-KO cells expressing exogenous human or elephant FANCL.
